# Editorial: Insights in Regulatory Science 2021

**DOI:** 10.3389/fmed.2022.1033558

**Published:** 2022-09-20

**Authors:** Bruno Sepodes, Peter Mol

**Affiliations:** ^1^Faculdade de Farmácia, Universidade de Lisboa, Lisbon, Portugal; ^2^Laboratory of Systems Integration Pharmacology, Clinical and Regulatory Science, Research Institute for Medicines (iMED.ULisboa), Lisbon, Portugal; ^3^Department of Clinical Pharmacy and Pharmacology, University Medical Center Groningen, University of Groningen, Groningen, Netherlands

**Keywords:** Regulatory Science, drug development, research and development, academia, regulatory agencies, Food and Drug Administration (FDA), European Medicines Agency (EMA)

Fast entering in the third decade of the 21st century, and still dealing with a challenging pandemic and other emerging health threats, anyone would agree that we are living unique times in drug development and regulation. Exceptional discoveries and advancements provide important inputs for the fast-growing domain of Regulatory Science. These pharmaceutical achievements are impacting the lives of millions of people all over the world but, should also be continuously reviewed and analyzed to ensure developments reach the market that bring efficacious and safe solutions to where they are most needed.

The acceleration of innovation is catalyzing the development of increasingly complex medicines, health products and medical devices, encompassing different and new technologies to promote, maintain and protect human health. To cope with these growing challenges, different regulatory agencies have established an open dialogue with stakeholders and prospectively planned strategies to enable developments in Regulatory Science that may be translated into better access to patients of new therapeutic opportunities in the everchanging landscape of health systems. In the Strengthening Training of Academia in Regulatory Science (CSA STARS) project European regulators engaged with academic drug researchers to improve translational success (1). Moreover, many efforts are being put forward on the improvement of evidence generation and quality of scientific assessments, on further collaborations with healthcare systems to promote patient access to medicines, and on the active management of new health threats, further integrating science and technology in medicines development.

Despite the known challenges in 2021, and the fight against the above-mentioned health threats—namely COVID-19—the year was marked by important advancements in new and innovative therapeutic options being brought to patients. Each year, a wide range of medicinal products for human use are approved. Some of these products have never been used in clinical practice and some represent “first in class” medicines, while others are similar or related to products who have previously been granted a marketing authorization. Additionally, in 2021, several extensions/variations to the original indication were granted, offering patients new uses for already existing medicines. Last year was an excellent year for the Food and Drug Administration (FDA) with a total of 60 new medicines approved (including 36 small molecules and 15 biologics) (2). Of notice, of these 60 new molecular entities approved by the FDA, 49 used incentives such as the ones deriving from the Orphan Drug Act or were approved under other schemes such as Priority, Fast-Track, Accelerated and/or Breakthrough designations (2). A close look into the European Medicines Agency (EMA) data shows that in 2021 the agency recommended 92 medicines for marketing authorization with some of them representing significant advancements in their therapeutic areas (3). Of these 92 medicines, three received a marketing authorization following an accelerated assessment, 13 received a recommendation for a conditional marketing authorization, four were authorized under exceptional circumstances and 19 had their orphan designation confirmed. Six of these medicines recommended for marketing authorization by the EMA had PRIME designation (an enhanced development support scheme provided by the EMA that “*aims at helping patients to benefit as early as possible from promising medicines that target an unmet medical need, by optimizing the generation of robust data and enabling accelerated assessment”*) (3). During 2021 another 14 medicines under development were included in PRIME (3). These continuous advancements in science, that translated into new therapeutic opportunities in 2021, go hand in hand with other developments namely in Regulatory Science.

With this Research Topic we wanted to capture some of the Regulatory Science advancements that are of relevance, focusing on new insights, novel developments, current challenges, latest discoveries, recent advances, and future perspectives in the field of Regulatory Science. Our goal was not only to shed light on the progress made in the past years, namely 2021, but also on some of the future challenges that Regulatory Science faces, providing a thorough overview of relevant topics carefully curated. We hope that this article collection will inspire, inform, and provide direction and guidance to researchers with an interest in Regulatory Science.

This Research Topic provides a unique mix of varied contributions aggregating 24 articles resulting from the work of 151 authors, and divided between two “*Brief Research Reports”*, nine “*Original Research”* articles, three “*Perspectives”* articles, six “*Policy and Practice Reviews”*, one “*Policy Brief”*, two “*Reviews”* and one “*Study Protocol”*. We took the editorial liberty of selecting a few of these works to spark the interest of readers and provide a substantiated glimpse of the state of the art in 2021.

Real world evidence continues to draw increasing attention between stakeholders worldwide, due to the potential supportive role in drug development and regulatory decision making. Li et al. discuss the experience of integrating Real-World Evidence in the Regulatory Decision-Making Process in the US, EU, and China. At the same time, Maeda and Ng add the perspective of Japan to this topic. One of the most downloaded original articles of this Research Topic brings us the work of Dekker et al., who assessed “*to what extent women were included in all phases of drug development; whether the clinical studies in the marketing authorization application dossiers include information per sex; and explored whether there are differences between women and men in the drugs' efficacy and safety”*. The assessment of sex proportionality in pre-clinical and clinical trials were performed in 22 applications for marketing authorization submitted to the European Medicines Agency (Dekker et al.). Several findings of this study are of interest to the readers of this Research Topic but the conclusion that the included number of women included in the studies was, however, not always proportional to disease prevalence rates is worth mentioning (Dekker et al.). These conclusions provide further guidance to those directly involved in the design of drug development.

There is also a chance to discuss the challenges of the Pediatric Regulation in Europe (Toma et al.), the results from the first multi-center European survey assessing the challenges in transition from childhood to adulthood care in rare metabolic diseases (Stepien et al.). Still in the topic of rare diseases and acknowledging the peculiarity of the definition of “Significant Benefit” introduced in the European Regulation for Orphan Medicinal Products in 2000, a reflection on the definition of “Satisfactory Methods of Treatment” relevant when assessing the Significant Benefit where the Regulators perspective is certainly extremely of special relevance. Since the notion of Significant Benefit is specific to the European Union regulation, it's important that stakeholders developing products for rare diseases are aware of these challenges here reported (Sheean et al.).

Other trending topics include the ongoing discussions on the use of biomarkers and companion diagnostics in drug development and how regulatory agencies are dealing with these developments (Orellana García et al.; Hendrikse et al.), the assessment and integration of patient preferences in assessing value in gene therapies (van Overbeeke et al.) and also, an in-depth analysis of the current landscape of implementation and access to Pre-exposure Prophylaxis for Human Immunodeficiency Virus by Men Who Have Sex With Men in Europe, that remains challenging and suboptimal despite available medicines and strategies (Sepodes et al.). HIV is also a pandemic we have been dealing with for many decades and traditional prevention strategies but also treatment as prevention are known available tools that could very well be the game changers we hope to see implemented worldwide, with the authors urging for further action in Europe (Sepodes et al.).

The Research Topic also dedicates special attention to developments in biologics (namely an important update on Biosimilars by Barbier et al.), genetically modified organisms (O'Sullivan et al.) and single-strain live biotherapeutic products entering First-in-Human Clinical Study, using feedback gained by EMA and FDA (Paquet et al.).

Given the challenges ahead, it won't come as a surprise that 2022 and 2023 will nurture further developments in Regulatory Science. Expectations remain high that we continue to be able to integrate these developments to ensure medical innovations translate more smoothly into the public health domain and address medical needs of patients around the world.

## Author contributions

BS and PM drafted the manuscript. All authors provided a critical revision of the manuscript, read, and approved the final manuscript.

## Conflict of interest

The authors declare that the research was conducted in the absence of any commercial or financial relationships that could be construed as a potential conflict of interest.

## Publisher's note

All claims expressed in this article are solely those of the authors and do not necessarily represent those of their affiliated organizations, or those of the publisher, the editors and the reviewers. Any product that may be evaluated in this article, or claim that may be made by its manufacturer, is not guaranteed or endorsed by the publisher.
